# Commentary: “Physical time within human time” and “Bridging the neuroscience and physics of time”

**DOI:** 10.3389/fpsyg.2023.1134397

**Published:** 2023-03-15

**Authors:** Natalja Deng

**Affiliations:** Underwood International College, Yonsei University, Seoul, Republic of Korea

**Keywords:** physical time, human time, temporal passage, illusion, temporal experience, local presentism, IGUS

Both Buonomano and Rovelli (2021) and Gruber et al. (2022) contain interesting interdisciplinary proposals for how to think about the relation between humans' experience of time and what time is like. This is a complex topic. Tackling it requires confronting difficult questions about (i) which features of experience and which features of time are difficult to fit together (if any), (ii) which discipline(s) should attempt the required explanation(s) (if any are required), and (iii) what these explanation(s) might look like. I'm very sympathetic to aspects of each proposal. In what follows, I offer some comments, starting with Buonomano and Rovelli (2021).

At the outset, Buonomano and Rovelli (hereafter B&R) distinguish three reasons why “the theoretical physicist is led to reject the idea that the commonsense view of time could remain valid outside a limited domain.” The first concerns the time reversal invariance of elementary mechanical laws, the second relativity's conflict with the notion of a global present, and the third the absence of a time variable in the basic equations of many theories of quantum gravity. They set aside the third as it pertains to the evolving frontiers of physics and concentrate on the first two, which pertain to well-established theories.

This is helpful, and it contrasts somewhat with the opening paragraphs of Gruber et al. (2022). My own philosophical disciplinary training would encourage putting the point here as follows: asking whether there is real passage (flow, becoming, and dynamicity) is different from asking whether time is real or fundamental, i.e., the block universe denies passage but not the reality of time. As it happens, my views are sufficiently unorthodox to make me hesitant about putting it this way (briefly: I wouldn't want to claim that the content of “passage is (not) real” is so discipline-transcendently clear that it is obvious from the outset how these two issues are to be neatly distinguished). But I do want to suggest that B&R's starting point is helpful and that it is not advisable, in one's exposition of “the two times problem” (Gruber et al., 2022), to equate claims about time not being fundamental or not existing with claims about there being no real passage. After all, B&R's first and second reasons relate more clearly to the latter, while the third relates more clearly to the former. And despite the elusiveness of big, unifying labels like “passage,” it helps to acknowledge that we're more concerned with passage and putative experiences thereof than with corresponding issues about time's (non)fundamentality.

So, what is the question? B&R describe it as “whether neuroscientists and physicists are talking about the same topic when they talk about time,” to which they answer “to some extent, “no,” [b]ut this may not necessarily mean there is an inconsistency.” At first sight, one might wonder about this juxtaposition of thoughts. Prima facie, if the two disciplines aren't talking about the same thing, that would seem to make it *less* likely that there is an inconsistency (compare: presumably psychotherapists and musicians are not referring to the same thing when they talk about the blues. Does that make it more or less likely that their claims about the blues might conflict?) Indeed, a reader new to the topic may wonder why it is necessary to “assign portions of sovereignty to the two fields,” when neuroscience studies the brain and physics the world (even if the world includes brains). But there is a perfectly good reason B&R say what they say, and I know of no other way to express it than to speak of *views of time*. If the two disciplines aren't talking about the same thing, that may indicate that the mind's view (picture, intuition) of time differs from the view of time suggested by the relevant physics. Put another way, different topics in the two disciplines may (or may not) suggest that there is some *content* to the way the brain is representing time that says something is the case which actually isn't, according to physics.

Putting it this way clarifies where B&R agree and disagree. For content like global presentism, they agree that (a) physics denies it, and probably also that (b) we represent it, if only in “intuition” (more cognitive aspects of temporal experience, perhaps a pre-theoretical view) or at least unschooled intuition. For content like static eternalism, they agree that (a) physics doesn't affirm or deny it, and (b) we don't represent it. So far, so good. Some intuitions have been proven wrong, but perhaps no deep illusion yet (affecting all aspects of temporal experience).

Now, what about content like local presentism? (a) Does physics deny it, and (b) do we represent it? Buonomano for one seems to answer “yes” to (b) (“irrepressible feeling”). As for (a), he probably answers “no,” but there is also textual evidence to the contrary. While he takes local presentism to be incompatible with the block universe, he doesn't think relativity implies the block universe; also, the local element is intended to sidestep conflicts with relativity. On the other hand, the interest in whether or not “closed timelike curves […] are a theoretical possibility” indicates an anticipated conflict with relativity, and presumably that conflict would go *via* some implied claim about the local past and future being as real as the present, i.e., the block universe again.

The way to clarify this further is to ask what (one thinks) the content of local presentism *is*, and whether (one thinks) it has a well-defined content at all (and corresponding questions for the other views). Both authors make comments that suggest important background commitments here, and without making those fully explicit, (a) and (b) are hard to tackle. In other words, a large part of the disagreement is in fact housed in metaontology.

Gruber, Block, and Montemayor (hereafter GBM) describe both B&R as “wanting to reify human time” so as not to posit a pervasive illusion. I take this to be a reaction to a feature of B&R's stances in Buonomano and Rovelli (2021), and especially Rovelli's (2019), which consists in a certain predilection for a very thorough kind of reconciliation, namely one that takes place at a meta-level. This predilection leads one to favor approaches to time that (somehow) transcend the dichotomy between dynamic and non-dynamic views, by (somehow) locating passage/flow/dynamicity *within* the block universe (I'm extremely sympathetic to these kinds of (“Tenseless Passage (TP)”) approaches^1^; I have also come to think TP still requires a philosophical foundation).

GBM's own approach shares some commonalities with B&R's, most notably in the claim that the block universe is “not ‘frozen’,” and relatedly, in the wish to build on the use to which authors like (Ismael, 2016) put Hartle's notion of an IGUS. However, GBM's overall approach, and their own use of the IGUS, is closer to that of Callender (2017), which (despite some of Callender's rhetoric) is more firmly rooted in the traditional distinction between dynamic and non-dynamic views of time. GBM see a clear explanatory gap between manifest and scientific time and are attempting to fill it.

For GBM, the key to reconciliation is to combine two principles, namely that (1) as an IGUS, the human “has an experience of past/present/future that is consistent with the physical laws” and that (2) “[t]he phenomenon of dynamism is an experimentally demonstrable illusory experience.” The resulting dualistic theory holds that there is a system producing veridical temporal experiences of the flow of time, but that this system also “begets a corresponding illusory system,” which is “the product of natural selection” and whose “sole purpose is to enhance the human experience of time.”

To interpret this, the first thing to ask is how the term “the flow of time” is being used and hence what exactly is at issue (see also the above comments on the opening paragraphs). On p. 4, GBM list the three most commonly associated ideas as “a unique (moving) present,” “dynamism of change/motion” and “directionality (temporality).” On p. 6, they mention “becoming” and say that while it should be recognized, it need not be “treated as a separate component of manifest time” because change has been dealt with in depth. One question I have here is what becoming involves that a moving present and dynamism don't, so that one can be set aside while the other two are accounted for. Another is whether the point about becoming is (a) that all we experience is (dynamic) change, without becoming, or (b) that all there is to the notion of becoming is contained in the notion of change, in which case an experience of change *is* an experience of becoming.

These questions have a direct bearing on how to interpret the dualistic theory. If, for instance, “becoming” denotes something similar to “dynamism” and the “moving” part of the moving present, and if these notions contain more than just the notion of change, then one would expect the dualistic theory to posit rather than deny illusory experiences of becoming alongside veridical experiences of change.

GBM do not dwell long on the distinction between perceptual and cognitive aspects of temporal experience (“the need to make a distinction between the terms cognitive and perceptual is not critical as Mroczko-Wasowicz […] questions the close relationship between the two”). Yet, manifest time is a multi-faceted phenomenon, and the stated aim is to account for “[a]ll major dualistic components of manifest time.” If nothing else, the distinction matters insofar as some pre-theoretical intuitions can be altered by schooling, while some aspects of perception cannot.

On p. 3, GBM claim that they are using the term “illusion” only because it is less cumbersome than “perceptual add-on,” citing phenomena in which the brain fills in missing information and guesses correctly. However, as they acknowledge, in those cases there is no mismatch between what is represented and what is the case, i.e., the perception is veridical. Yet, in GBM's exposition of the dualistic theory, the terms “illusory” and “veridical” both play a central role, and it would be puzzling if they there meant the same thing. Moreover, GBM's suggestion that when “only cognition is involved such as a myth or belief it can be referred to as a cognitive add-on” adds to the puzzle, because a myth does suggest a mismatch between representation and reality.

While the “perceptual add-on” terminology seems intended to soften some of the original implications of the term “illusion,” this is probably not helpful to a defender of the dualistic theory. The idea has to be that the first principle posits some (perceptual and/or cognitive) veridical aspects of temporal experience, and that the second principle posits some (perceptual and/or cognitive) illusory aspects of temporal experience, where these come in pairs. The illusory component of each of these pairs involves perceptual experiences as of x and/or a belief that x is the case, where x does not exist and is not the case, according to physics.

As a final illustration, consider GBM's stance on persistence. Miller and Wang (2022) helpfully point out that the block universe may well be compatible with endurantism and that perdurantism is in any case also a view of persistence. They further conjecture that GBM's view may be that there is no (unchanging core) persisting self. This seems likely, and it too strongly suggests reliance on the usual meaning of “illusory,” because the idea is likely to be that while we don't persist, we seem to and/or ordinarily think we do. Thinking of the sense of a persisting self as a mere “add-on” in the sense of a filling in of information that turns out to be a correct guess would not fit with insisting that there isn't one, in reality.

## Author contributions

The author confirms being the sole contributor of this work and has approved it for publication.

## Footnotes

^1^Examples of TP views include, but are probably not limited to (here listing only one work per author) Savitt (2002), Dieks (2006), Dorato (2006), Maudlin (2007), Harrington (2009), Deng (2013), Mozersky (2015), Oaklander (2015), Fazekas (2016), Ismael (2016), Arthur (2019), Rovelli (2019), Saudek (2020), Leininger (2021).
